# A data-informatics method to quantitatively represent ternary eutectic microstructures

**DOI:** 10.1038/s41598-018-37794-y

**Published:** 2019-02-07

**Authors:** Irmak Sargin, Scott P. Beckman

**Affiliations:** School of Mechanical and Materials Engineering, Washington State University, Pullman, Washington 99164 USA

## Abstract

Many of the useful properties of modern engineering materials are determined by the material’s microstructure. Controlling the microstructure requires an understanding of the complex dynamics underlying its evolution during processing. Investigating the thermal and mass transport phenomena responsible for a structure requires establishing a common language to quantitatively represent the microstructures being examined. Although such a common language exists for some of the simple structures, which has allowed these materials to be engineered, there has yet to be a method to represent complex systems, such as the ternary microstructures, which are important for many technologies. Here we show how stereological and data science methods can be combined to quantitatively represent ternary eutectic microstructures relative to a set of exemplars that span the stereological attribute space. Our method uniquely describes ternary eutectic microstructures, allowing images from different studies, with different compositions and processing histories, to be quantitatively compared. By overcoming this long-standing challenge, it becomes possible to begin to make progress toward a quantitatively predictive theory of ternary eutectic growth. We anticipate that the method of quantifying instances of an object relative to a set of exemplars spanning attribute-space will be broadly applied to classify materials structures, and may also find uses in other fields.

## Introduction

Materials design involves observing and cataloging materials structures, understanding the underlying relationship between the multilevel structures and resulting material properties, and developing processing routes to prepare materials with the properties that yield optimal engineering performance^1^. It relies upon the existence of a universally agreed upon language to quantitatively represent and subsequently catalog the observed structures^2^. One of the most fundamental components of a material’s hierarchical structure is the microstructure; however, there is yet to be a consensus regarding the language for quantitatively representing many complex microstructures.

Recent attempts at microstructural quantification have involved data-driven and machine learning based approaches^3–7^. Principal component analysis (PCA), a statistical method that has been successfully applied to identify trends in complex multivariate materials data^8–10^, has also found application in the quantitative representation of microstructures. PCA was used by Zabaras *et al*. to construct a dynamic library of single phase polyhedra microstructures^11,12^. Instead of focusing on the many sub-features of the microstructures, each was considered as a single entity that was quantified by a set of coefficients. Single grain microstructures were used as input to PCA and were combined with a support vector machine algorithm for classification^11,12^. PCA also has been used in stochastic modeling of microstructures. The spatial relations were described using two-point correlations and the overall state of the structure was treated as a statistical distribution^2,13–16^. The variance of the two-point correlations was examined in PCA and this allowed the creation of a structure-property map in PCA space^2,13^. This approach allows the simultaneous classification and structure-property analysis of multiple complex microstructures^14^ including, for example, the structure-diffusivity relationship in porous transport layers of polymer electrolyte fuel-cells^15^, and the structure-plasticity relationship in non-metallic inclusion/steel composite material systems^16^. A similar method has been used to couple phase field simulations with spatial correlations to quantify and classify the evolution of microstructural changes in ternary eutectic structures^17,18^.

In this work we also focus on the ternary eutectic microstructures, as an exemplar. Even simple ternary microstructures exhibit a high degree of morphological variation due to the complex dynamics present during their evolution. The significant advances that have been made in understanding the solidification of regular binary eutectic compounds^19,20^ are based on the common language used to quantitatively describe the resulting microstructure, *i*.*e*., the lamellar and rod morphologies. The absence of a universal language to quantitatively represent the ternary eutectic microstructures has prohibited the development of an accurate theory of ternary eutectic solidification and this motivates our study.

Initial classification efforts described ternary eutectic microstructures as a combinations of the lamellar and rod morphologies observed in the regular binary eutectic microstructures^21–23^, but this approach was unable to represent the multitude of complex morphologies observed in experiments^24–28^. Currently, the most widely used classification scheme for ternary eutectic morphologies is given by Ruggiero and Rutter^29^ and its analytical solution is an extension of Jackson and Hunt’s analytical solution of binary eutectics^30^; three distinct growth modes are identified: semi-regular brick (SRB), lamellar (LAM), and rod-hexagon (RHN)^31,32^. A small set of geometric features, such as fixed eutectic spacings and the fixed spacing ratio of phases are used to describe the relative scale of these microstructures. These approaches, although yielding important insights, have yet to produce a universal representation of ternary eutectic microstructures that can be used to develop a predictive model of solidification. As a result, new parameters have been suggested to describe the microstructures^33,34^. The greatest challenge for developing a quantitatively correct theory of ternary eutectic solidification is the creation of a universal language to allow a representation of the observed structures.

We demonstrate a data informatics approach to quantitatively represent the ternary eutectic microstructure. The microstructures examined are from the ternary eutectic Al-Cu-Ag system and are taken from refs^35,36^, along with the stereological descriptors used to describe them. Although there is a continuum of possible structures, the data-informatics method presented here allows any microstructure to be uniquely referenced to the three idealized morphologies identified in refs^29,31,32^. This approach directly allows for the integration of data spanning sources. In addition, the resulting numerical regression is applicable to ternary eutectic microstructures of other compositions.

## Results

### Dataset

**Figure 1:**
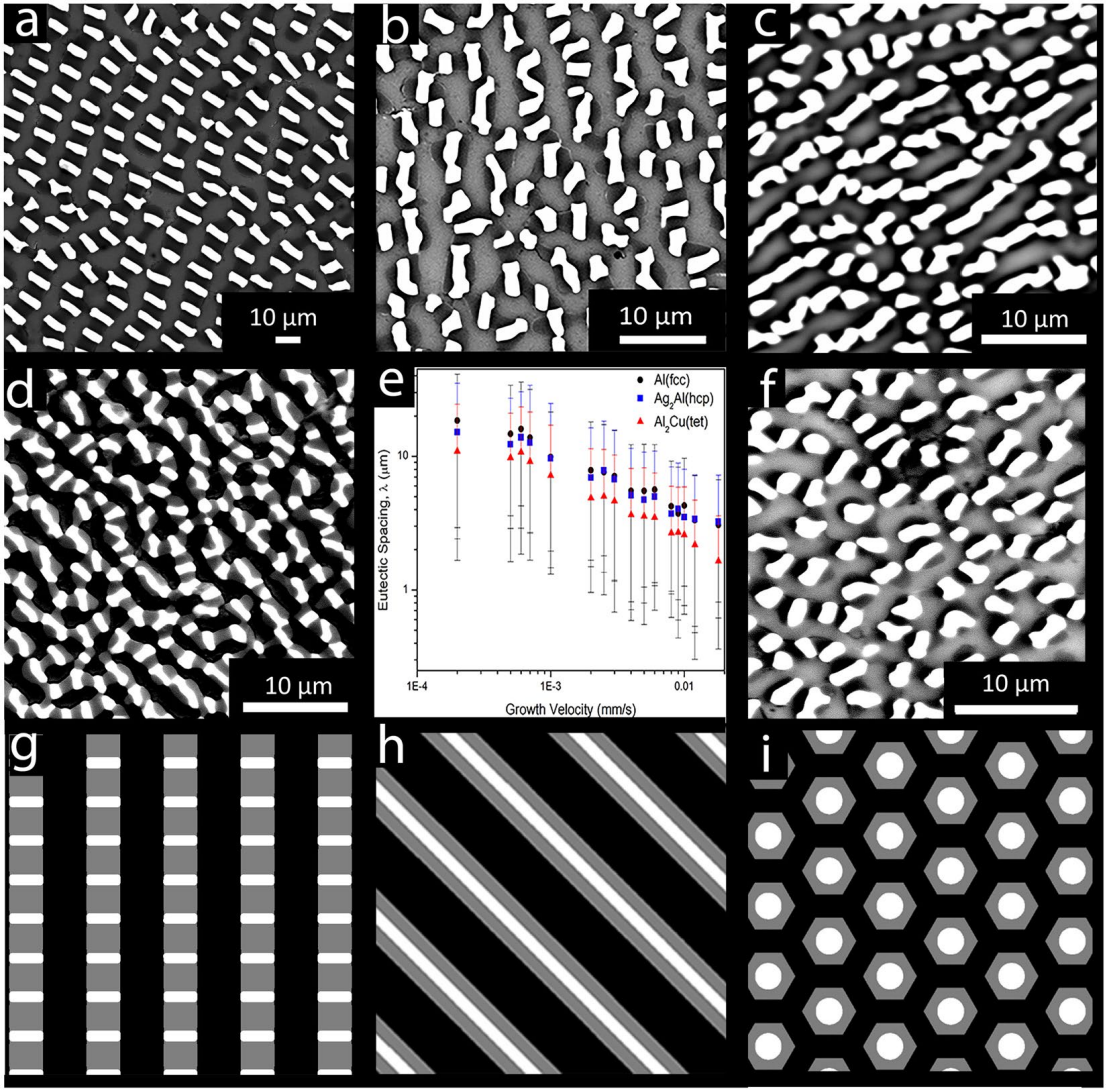
Summary of the previous work. (**a**–**d**) Representative microstructures observed for directionally solidified ternary eutectic Al(fcc), Ag_2_Al(hcp), and Al_2_Cu(tet) for a variety of velocities from ref.^35^: (**a**) 0.0006 mm/s; (**b**) 0.004 mm/s; (**c**) 0.010; and (**d**) 0.012 mm/s;. For (**a**–**c**) the black phase is Al_2_Cu(tet), Al(fcc) is gray, and Ag_2_Al(hcp) is white. For (**d**) Al_2_Cu(tet) and Al(fcc) are reversed with Al_2_Cu(tet) being gray and Al(fcc) black. (**e**) The phase spacing as a function of velocity^35,36^. (**f**) The microstructure observed at 0.009 mm/s. (**g**–**i**) The ideal ternary eutectic microstructures as described in refs^29,31,32^: (**g**) semi-regular brick (SRB); (**h**) lamellar (LAM); and (**i**) rod-hexagon (RHN). Al(fcc), Ag_2_Al(hcp), and Al_2_Cu(tet), are assigned the pixel values 2, 255, and 127 respectively.

It was reported in refs^35,36^ that three variations of the semi-regular brick structure were observed in samples produced by directional solidification processed over the velocity range 0.0005 mm/s to 0.018 mm/s. In the low-velocity range, 0.0005 mm/s to 0.001 mm/s, an ordered semi-regular brick structure was observed; at mid-velocity range, 0.001 mm/s and 0.01 mm/s, the intermetallic phases, Ag_2_Al(hcp) and Al_2_Cu(tet), began connecting to their nearest same phase neighbors resulting in a more elongated form; at higher velocities the microstructure somewhat resembled the lamellar morphology. Due to the relatively slow diffusion rate of Ag in liquid, at high velocities the Ag_2_Al(hcp) phase had a more fragmented morphology as compared to the Al_2_Cu(tet) phase, which continued to maintain a lamellar form. At the highest velocities Al(fcc) lost its elongated form, adopting a more circular shape, similar to Ag_2_Al(hcp), and the periodicity between intermetallic phases also became less regular. The morphological changes in the images were smooth with increasing velocity. Examples of these microstructures are shown in Fig. 1.

These observations, while instructive for understanding the Al(fcc)-Ag_2_Al(hcp)-Al_2_Cu(tet) ternary eutectic system, do not provide a quantitative representation that allows the development of a general theory of solidification, nor do they allow for comparison of these microstructures to those reported from other studies. Well-known stereological descriptors that define the scale of the phases provide a quantification of the microstructure, but do not accurately capture all the qualitative changes observed in the images^35^. For example, Fig. 1(e) shows that the relative eutectic spacing remains constant for all growth velocities. This quantitative result does not explain the apparent microstructural differences observed in Fig. 1(a–d). Sargin and Napolitano concluded that scale defining attributes alone are insufficient to quantitatively represent the microstructures and developed descriptors that describe both the shape and scale of the phases^35,36^.

The stereological attributes used in the data analysis, consist of three different elements. The first element involves the quantification of area, perimeter, length, and width of each phase. The phase fractions are obtained from area measurement. The values are then averaged across each image. The second element is analysis of Fourier transform patterns from single-phase masked images. For each Fourier transform pattern, a radial and angular distribution plot is generated. The radial and angular order parameters are defined as the height over width of the peaks in the distribution plots. The final element is the analysis of the phase boundary distribution according to the angle and type. The 19 stereological attributes used in this study are given in Table 1.

**Table 1:** The stereological attributes used here and described in ref.^35^.

Abbreviation	Attribute
L/W1	Length over width ratio of Al(fcc) in log_10_
L/W2	Length over width ratio of Ag_2_Al(hcp) in log_10_
L/W3	Length over width ratio of Al_2_Cu(tet) in log_10_
A/P1	Area over perimeter ratio of Al(fcc) in natural log_10_
A/P2	Area over parameter ratio of Ag_2_Al(hcp) in log_10_
A/P3	Area over parameter ratio of Al_2_Cu(tet) in log_10_
SF1	Shape factor (Area^2^/4*π* Area) of Al(fcc) in log_10_
SF2	Shape factor (Area^2^/4*π* Area) of Ag_2_Al(hcp) in log_10_
SF3	Shape factor (Area^2^/4*π* Area) of Al_2_Cu(tet) in log_10_
AO1	Angular Order of Al(fcc) in log_10_
AO2	Angular Order of Ag_2_Al(hcp) in log_10_
AO3	Angular Order of Al_2_Cu(tet) in log_10_
RO1	Radial Order of Al(fcc) in log_10_
RO2	RadialOrder of Ag_2_Al(hcp) in log_10_
RO3	Radial Order of Al_2_Cu(tet) in log_10_
PF1	Phase Fraction of Al(fcc)
PF2	Phase Fraction of Ag_2_Al(hcp)
PF3	Phase Fraction of Al_2_Cu(tet)
APB	Orientation difference between Al(fcc)/Al_2_Cu(tet) and Al_2_Cu(tet)/Ag_2_Al(hcp) phase boundaries in degrees

Rather than compare microstructures to each other directly, a quantitative representation is constructed that describes the microstructures relative to the ideal SRB, LAM, and RHN ternary eutectic microstructures proposed by Ruggiero and Rutter^29,31,32^. The ideal microstructural images with equilibrium phase fractions, shown in Fig. 1(g–i), are generated such that the scale of the microstructures are consistent with that of the sample pulled at 0.001 mm/s. The phases are assigned the same pixel values as the masked experimental ones, 2, 255, and 127 for Al(fcc), Ag_2_Al(hcp), and Al_2_Cu(tet), respectively. The stereological attributes extracted for these three are combined with data from refs^35–37^, discussed above, to yield the dataset.

### Data analysis

**Figure 2:**
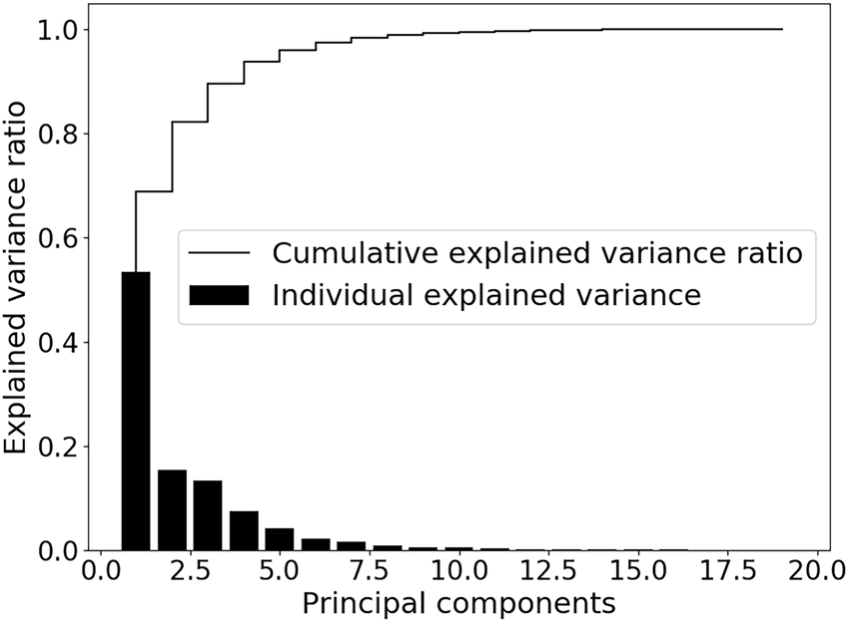
The individual and cumulative explained variances.

**Figure 3:**
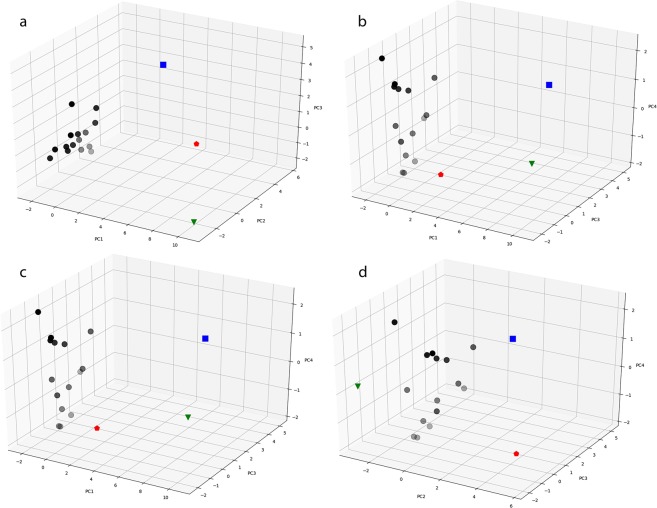
The PC scores of microstructures shown for the first four PCs: (**a**) PC1, PC2, and PC3; (**b**) PC1, PC2, and PC4; (**c**) PC1, PC3, and PC4; (**d**) PC2, PC3, and PC4. The gray scale symbols are the experimental microstructures, the blue square is the ideal SRB, the green upside down triangle is the ideal LAM, and the red polygon is the ideal RHN.

The dataset is standardized and PCA is performed. The scree plot in Fig. 2 shows that 90% of the variance in the dataset is accounted for by the first four principal components (PCs). The loadings of these PCs are given in Table 2 and the score plots are given in Fig. 3.

**Table 2:** Loadings of the first four PCs.

Attribute	PC1	PC2	PC3	PC4
L/W1	0.506	−0.335	0.700	0.024
L/W2	0.631	−0.794	0.050	0.033
L/W3	0.564	−0.554	−0.626	−0.043
A/P1	−0.835	0.387	−0.402	−0.130
A/P2	−0.894	0.280	−0.383	−0.088
A/P3	−0.953	−0.043	−0.261	−0.003
SF1	−0.376	0.883	−0.089	−0.189
SF2	−0.674	−0.468	0.507	−0.072
SF3	−0.720	−0. 231	0.380	−0.307
AO1	0.823	−0.129	−0.481	−0.004
AO2	0.884	−0.340	−0.257	−0.093
AO3	−0.904	−0.151	−0.309	−0.088
RO1	0.869	0.435	0.050	0.007
RO2	0.936	0.158	0.219	0.086
RO3	0.982	0.132	0.053	0.143
PF1	0.791	0.290	0.191	−0.510
PF2	−0.870	−0.427	−0.145	−0.039
PF3	−0.089	0.136	−0.123	0.997
APB	0.005	0.318	0.701	0.198

The distinction between experimental and ideal microstructures is the source of greatest variance in the dataset, as shown in Fig. 3(a–c), and is captured by PC1. In contrast, the score plot of PC2, PC3, and PC4, in Fig. 3(d), shows that the experimental microstructures are bracketed by the ideal SRB, LAM, and RHN structures. The loadings of PC2, PC3, and PC4 have a larger variability that PC1 demonstrating that each PC represents unique stereological information. PC1 is removed for the remainder of the analysis due to its primarily characterizing the trivial distinction between the experimental and ideal structures.

**Figure 4:**
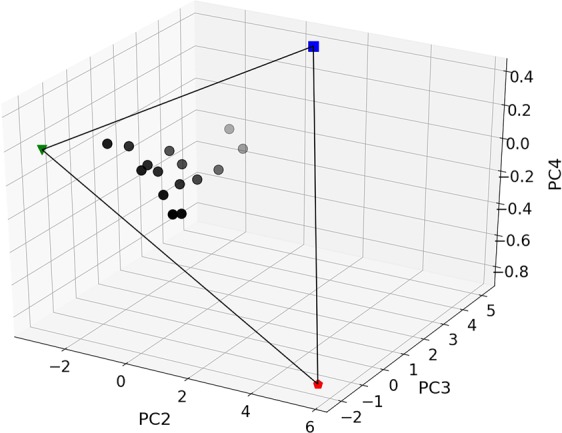
The PC scores projected onto the plane defined by the SRB, LAM, and RHN structures in PC-space defined by PC2, PC3, and PC4. The gray scale symbols are the experimental microstructures, the blue square is the ideal SRB, the green upside down triangle is the ideal LAM, and the red polygon is the ideal RHN.

**Figure 5:**
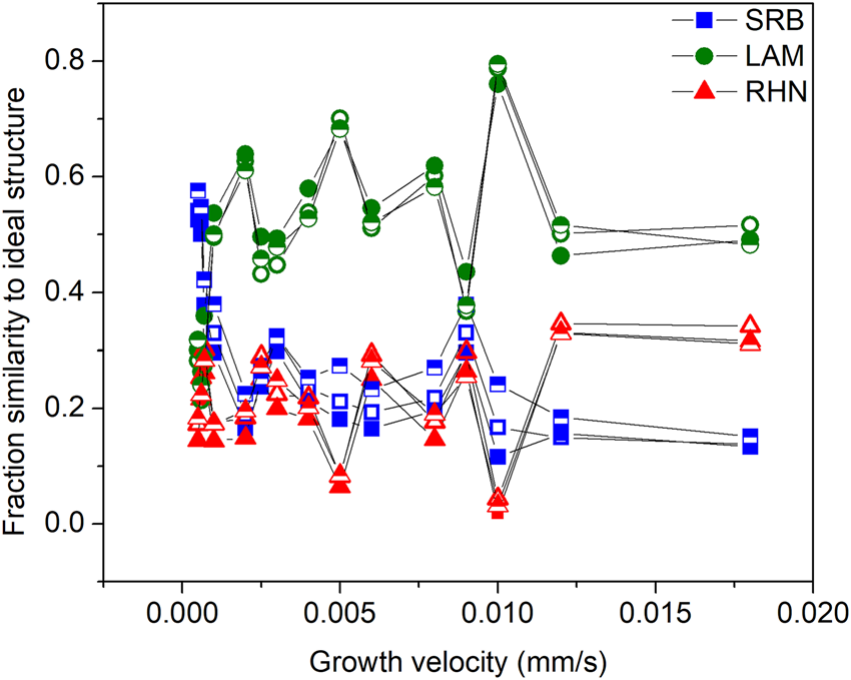
The fraction similarity to SRB, LAM, and RHN ploted versus solidification velocity. Filled symbols are fractions obtained from triangulated values in PCA, the empty symbols are the PLS regression results using all 19 attributes, and half-filled symbols show the results from a 9 attribute PLS regression.

The principal component transformation is distance preserving, *i*.*e*., the relative distances between samples in attribute space remain the same in PC space, therefore it is possible to use the relative distance between experimental and ideal microstructures in PC space to quantify the similarity of experimental microstructures to the ideal ones. The experimental scores are projected into the plane defined by the SRB, LAM, and RHN scores, as shown in Fig. 4. This allows each experimental microstructure to be uniquely triangulated in terms of its fraction similarity to the ideals. The fraction similarity to the SRB, LAM, and RHN structures is determined for each microstructure and the results are plotted in Fig. 5. The growth velocity is used to label each microstructure in the figure.

A partial least squares (PLS) regression is used to quantify the relationship between a microstructure’s stereological attributes and its fraction similarity to the ideal structures. To cross validate the regression, the leave-one-method is used. This approach helps compensate for the relatively small sample population. The resulting regression has a mean squared error (MSE) of 4.8 × 10^−5^, 7.5 × 10^−5^, and 1.6 × 10^−5^ for SRB, LAM, and RHN, respectively.

It is known from the PCA that not all 19 of the stereological attributes are significant and it is found that using only 9 attributes yields a regression with MSE 1.4 × 10^−3^, 6.0 × 10^−4^, and 4.0 × 10^−4^ for SRB, LAM, and RHN, respectively. Reducing the number of attributes allows the most significant ones to be identified and assists future studies by reducing the stereological analysis needed. The predicted regression results using only 9 attributes are compared to regression results using all 19 attributes in Fig. 5. The regressions are written

$$\begin{array}{rcl}{\rm{SRB}} & = & \sum _{n=1}^{n=9}\,{C}_{{\rm{n}}}{A}_{{\rm{n}}}-0.2646,\\ {\rm{LAM}} & = & \sum _{n=1}^{n=9}\,{C}_{{\rm{n}}}{A}_{{\rm{n}}}+0.9573,\\ {\rm{RHN}} & = & \sum _{n=1}^{n=9}\,{C}_{{\rm{n}}}{A}_{{\rm{n}}}+0.3493,\end{array}$$

where *A*_*n*_ are the measured attributes and *C*_*n*_ are the coefficients given in Table 3.

**Table 3:** The attributes and coefficients for the PLS regression of SRB, LAM, and RHN.

Semi-regular brick					Lamellar				Rod-hexagon			
n	Att.	*β*_*n*_	*σ*_*n*_	*C*_*n*_	Att.	*β*_*n*_	*σ*_*n*_	*C*_*n*_	Attr.	*β*_*n*_	*σ*_*n*_	*C*_*n*_
1	APB	0.0794	0.0050	0.0021	APB	−0.0881	0.0092	−0.0023	SF1	0.0676	0.0034	0.4368
2	AO1	−0.0545	0.0035	−0.0749	RO1	−0.0691	0.0077	−0.2744	L/W1	−0.0551	0.0033	−0.2753
3	SF2	0.0516	0.0040	0.1447	SF1	−0.0678	0.0085	−0.4384	RO1	0.0426	0.0034	0.1693
4	L/W1	0.0468	0.0045	0.2338	AO2	0.0583	0.0093	0.1270	P/A1	0.0396	0.0028	0.0434
5	P/A2	−0.0389	0.0038	−0.0619	PF2	0.0565	0.0065	0.9054	L/W2	−0.0390	0.0022	−0.2258
6	P/A1	−0.0342	0.0033	−0.0374	AO1	0.0551	0.0063	0.0757	SF2	−0.0368	0.0027	−0.1031
7	AO2	−0.0331	0.0051	−0.0721	PF1	−0.0462	0.0044	−0.6310	PF2	−0.0310	0.0033	−0.4973
8	L/W3	−0.0331	0.0055	−0.1805	P/A3	−0.038	0.0102	−0.0581	P/A3	0.0308	0.0055	0.0470
9	SF3	0.0328	0.0019	0.1021	AO3	0.0260	0.0059	0.0537	PF1	0.0266	0.0022	0.3630

Here *β*_*n*_ is standardized coefficient and *σ*_*n*_ the standard deviation. *C*_*n*_ is the coefficient used in the PLS regression. In this table the attributes are listed in order of decreasing importance.

## Discussion

It has been shown that ternary eutectic microstructures cannot be quantitatively represented using simple geometric measures, nor can they be represented using stereological analysis of individual features^35^. Here we demonstrate that it is possible to decompose ternary eutectic microstructures using stereological attributes and then apply informatics methods to quantify the similarity of the microstructure relative to the ideal SRB, LAM, and RHN structures.

The directionally solidified microstructures’ fractional similarity to the ideal LAM, SRB, and RHN structures is shown in Fig. 5. Analysis of the results tells us that for velocities less than 0.001 mm/s the structures are strongly represented as SRB; for example, the microstructure produced by solidifying at 0.0006 mm/s is determined to have a structural character of (0.55/0.25/0.20) (SRB/LAM/RHN). Increasing the solidification velocity results in a reduction of SRB character and increase in the LAM character. For velocities ranging between 0.0025 and 0.010 mm/s the average character is (0.21/0.58/0.21). At higher velocities the microstructure becomes a mixture of the LAM and RHN features, and at a velocity of 0.012 mm/s the character is (0.20/0.45/0.35). These quantitative predictions are in excellent agreement with the features qualitatively observed in Fig. 1: Fig. 1(a) demonstrates SRB features for low velocities, Fig. 1(b,c) demonstrate a primarily LAM structure, and Fig. 1(d) demonstrates a mixing of the LAM and RHN features.

Looking closely at the results in Fig. 5 we see a large change in predicted character going from 0.009 mm/s to 0.010 mm/s. Such a large change in microstructure for an incremental change in velocity is unexpected, but careful examination of the 0.009 mm/s structure, shown in Fig. 1(f), and the 0.010 mm/s, shown in Fig. 1(c), validates this prediction. Although at this time it is unclear the physical origin of the changes, it is apparent that our analysis method accurately and quantitatively captures them.

Our confidence in this approach to represent the microstructures is due to the overall robustness of the analysis methods and data. First, PCA is a distance preserving transformation, meaning that the relative stereological similarities of the samples in attribute space is preserved when transformed to PC space. The first four PCs capture 90% of the attribute variance, meaning that the resolution loss due to truncation is on the order of the uncertainty introduced from other sources, such as the inherent variability from the experimental methods. Second, the first PC clearly captures the large difference between the ideal and experimental images. This is immediately observable from the score plots in Fig. 3(a–c). Discarding the trivial information from PC1 refines the data, leaving the important differences in the remaining PCs. This is analogous to masking the through beam in an electron diffraction experiment allowing access to the fine grained information in the diffracted data. Third, even though only a limited number of images are available, each image is information rich and the stereological analysis makes redundant measurements across each image. The attributes used in the analysis are not single measurements, but are averages from 25 different images taken from each sample. Fourth, this method is able to quantify the dramatic changes in the microstructures as the solidification velocities change, for example the crossover between SRB and LAM at 0.001 mm/s. The emergence of RHN at velocities greater than 0.012 mm/s also is clearly demonstrated. Fifth, the method presented here is purely based on experimental microstructures, comparing them to a small set of idealized archetypal structures. It does not require any theoretical inputs that may bias the results.

The PLS regression can be used for determination of fraction similarity to SRB, LAM, and RHN of any invariant ternary eutectic structure, independent of alloy system, *i*.*e*., the attributes have no explicit compositional dependence. The regression also is independent of the velocity and thermal gradient because the ideal standards used in the mapping do not depend on processing.

## Conclusions

An approach is demonstrated that accurately and quantitatively represents ternary eutectic microstructures by classifying them according to their relative similarity to the ideal SRB, LAM, and RHN microstructures. The method is sufficiently general that it can be applied to any directionally solidified invariant ternary eutectic microstructure regardless the alloy system. Therefore it provides the common language necessary for the development of a general theory of ternary eutectic microstructure evolution during directional solidification.

Although this study is focused on microstructure, the data analysis process can be applied to quantitatively characterize any collection of structures or objects that have well-defined bracketing ideals. The essential elements of the approach are the identification of ideal standards, the identification of quantifiable attributes, the use of the attributes and PCA to classify the structures, the determination of the relevant PCs needed to explain the sample variance, and the exclusion of the trivial PCs that merely highlight the difference between the ideal and actual structures.

## Methods

In the dataset, the length over width, area over perimeter, shape factor, angular order, and radial order attributes were subjected to a logarithmic transformations. Attribute standardization was used to avoid bias due to scaling differences. Standardization was applied according to the equation,

$${X}_{S}^{i}=({X}^{i}-{\mu }_{X})/{\sigma }_{X},$$

where ${X}_{S}^{i}$ was the standardized value of the data set, *μ*_*x*_ was the mean and *σ*_*X*_ was the standard deviation.

Following this, PCA was applied and the distances and similarities of experimental structures to the ideal ones were calculated. The microstructures were characterized relative to the ideal ones by projecting them into the plane defined by the SRB, LAM, and RHN structures in PC2-PC3-PC4-space. The PC1 component was discarded because it primarily contained the information distinguishing the experimental and ideal images. The projected microstructures were bound by a triangle defined by the SRB, LAM, and RHN, which allowed the similarity of each microstructure to be determined in terms of its unique position relative to the ideal structures.

A PLS regression was used to determine the microstructures’ fraction similarity to SRB, LAM, and RHN as a function of the microstructural attributes. The scikit-learn’s^38^ PLS package with the nonlinear iterative partial least squares (NIPALS) algortihm was used. The optimal number of components to be used in the PLS regression was obtained by the leave-one-out cross-validation method. For SRB and RHN 8 PCs were used and for LAM 9. The minimum number of attributes needed to accurately represent the triangulated data was determined to be 9. The 9-attribute linear equation for SRB, LAM, and RHN, were determined independent of each other.
